# A Motion Intention Recognition Method for Lower-Limb Exoskeleton Assistance in Ultra-High-Voltage Transmission Tower Climbing

**DOI:** 10.3390/s26082346

**Published:** 2026-04-10

**Authors:** Haoyuan Chen, Yalun Liu, Ming Li, Zhan Yang, Hongwei Hu, Xingqi Wu, Xingchao Wang, Hanhong Shi, Zhao Guo

**Affiliations:** 1 State Grid Hubei Electric Power Co., Ltd., Exta High Voltage Company, Wuhan 430050, China; 2 School of Robotics, Wuhan University, Wuhan 430072, China

**Keywords:** transmission tower climbing, lower-limb exoskeleton, motion intention recognition, bidirectional long short-term memory (Bi-LSTM), temporal attention mechanism

## Abstract

Transmission tower climbing is a critical specialized operation in ultra-high-voltage power maintenance and communication infrastructure servicing. However, existing lower-limb exoskeletons used for tower climbing still suffer from insufficient motion intention recognition accuracy under complex operational environments. To address this issue, this study proposes an inertial measurement unit (IMU)-based bidirectional temporal deep learning method for motion intention recognition. First, a one-dimensional convolutional neural network (1D-CNN) is employed to extract local temporal features from multi-channel IMU signals. Subsequently, a bidirectional long short-term memory network (Bi-LSTM) is introduced to model the forward and backward temporal dependencies of motion sequences. Furthermore, a temporal attention mechanism is incorporated to emphasize discriminative features at critical movement phases, enabling the precise recognition of short-duration and transitional motions. Experimental results demonstrate that the proposed method outperforms traditional machine learning approaches and unidirectional temporal models in terms of accuracy, F1-score, and other evaluation metrics. In particular, this method demonstrates significant advantages in identifying the flexion/extension phases and transitional states. This study provides an offline method for analyzing movement intentions in lower-limb exoskeleton control for power transmission tower climbing scenarios and offers a reference for developing assistive control strategies for assisted climbing tasks in this specific context.

## 1. Introduction

With the large-scale construction of power and communication infrastructures, transmission tower climbing has become a typical high-risk and high-intensity industrial task for transmission line workers [1,2]. During operation, workers are required to carry various tools and equipment while performing repetitive lower-limb climbing actions, including stepping, lifting, supporting, and transitioning between ladder rungs. Long-term engagement in such tasks may lead to physical fatigue and even operational accidents [3,4]. Therefore, lower-limb assistive exoskeletons have attracted widespread attention as an effective solution to reduce physical burden and improve operational safety in specialized working environments [5,6]. However, in practical scenarios, lower-limb exoskeleton robots must achieve a high level of human–robot coordination in dynamic and unstructured environments. Their control performance largely depends on accurate and real-time perception and recognition of human motion states and intentions [7].

For exoskeleton-assisted climbing tasks, accurate sensing and recognition of human motion intention are prerequisites for achieving safe and efficient human–robot collaboration. Current mainstream sensing modalities include electromyography (EMG) and inertial measurement units (IMUs) [8]. EMG captures muscle electrical signals to perceive pre-movement intention; however, in transmission tower climbing operations, electrodes are prone to detachment due to sweating and exhibit weak resistance to electromagnetic interference, making them unsuitable for complex outdoor environments. In contrast, inertial measurement units (IMUs), owing to their small size, low power consumption, and flexible deployment, have become the most commonly used wearable sensors in exoskeleton systems for continuously acquiring acceleration, angular velocity, and orientation data [9,10,11]. Zhang et al. used historical IMU readings to predict the three-dimensional posture of a human in future frames for a method of predicting lower-limb hip joint posture, thereby compensating for system latency [12].

With the rapid development of artificial intelligence, machine learning and deep learning methods have been increasingly introduced into IMU-based motion recognition. Traditional approaches rely on handcrafted features combined with classifiers such as support vector machines (SVMs) [13] or k-nearest neighbors (KNNs) [14], but they often suffer from limited generalization capability in complex industrial environments. Deep neural networks—particularly convolutional neural networks (CNNs) [15] and recurrent neural networks (RNNs) [16]—have demonstrated superior capability in learning discriminative spatiotemporal features directly from raw IMU signals. Hybrid CNN–LSTM architectures have thus become a common choice for wearable motion analysis [17,18]. Semwal et al. compared artificial neural networks, extreme learning machines and CNN-based deep neural networks by configuring IMU sensors to analyze different joint patterns in human gait; the results indicated that the CNN-based deep neural network achieved the highest classification accuracy [19]; however, most existing studies adopt unidirectional temporal modeling, which may fail to fully exploit the complete temporal context of periodic and transitional lower-limb movements during tower climbing [20,21,22].

To further enhance the robustness and discriminative capability of IMU-based motion intention recognition, a bidirectional long short-term memory network (Bi-LSTM) is introduced [23]. By simultaneously modeling forward and backward dependencies in time series, Bi-LSTM can more comprehensively capture the dynamic evolution of motion patterns within a full temporal window, making it particularly suitable for periodic and phase-dependent lower-limb movements. In addition, a temporal attention mechanism [24] assigns adaptive weights to features at different time steps, enabling the model to focus on key segments relevant to motion intention recognition. This mechanism helps mitigate noise interference and improves sensitivity to short-duration motion states.

Based on the above analysis, this paper proposes a Bi-LSTM network embedded with a temporal attention mechanism for motion intention recognition of lower-limb exoskeletons in transmission tower climbing scenarios. The proposed method efficiently extracts spatiotemporal features from multi-IMU sequential data and achieves accurate recognition of complex motion patterns. While ensuring strong representational capability, the method also considers real-time requirements, providing a foundation for assistive control of exoskeleton systems.

## 2. Materials and Methods

### 2.1. Description of the Transmission Tower Climbing Operation Scenario

In transmission tower climbing scenarios, maintenance personnel are required to carry toolkits and perform high-altitude operations while repeatedly executing lower-limb movements, such as extension, flexion, and alternating legs between rungs, as defined in Table 1.

From a kinematic perspective, tower climbing movements exhibit distinct periodicity, frequent short-duration states, and rapid transitions. Compared to everyday walking and stair climbing, the joint angles and accelerations of hip and knee flexion and extension are markedly different; the hip angle in the sagittal plane can reach up to 140°, whereas it is only 40° during walking; furthermore, working at heights inherently carries certain risks, which poses a significant challenge for the rapid and accurate recognition of movement intentions by lower-limb exoskeleton robots designed to assist in tower climbing [25].

**Figure 1:**
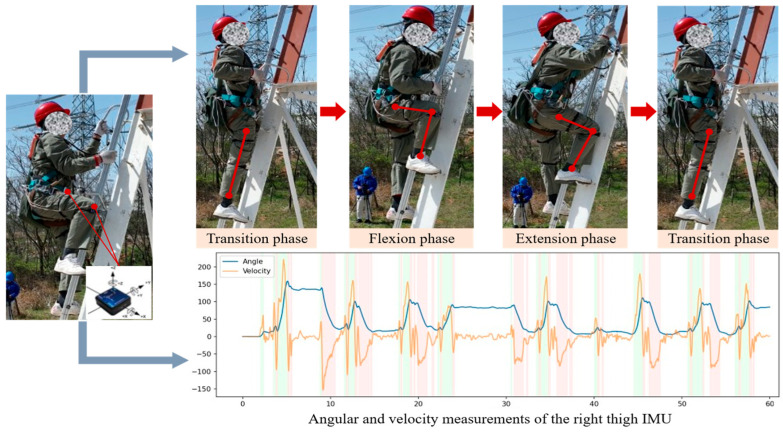
Motion posture data acquisition and analysis during ultra-high-voltage transmission tower climbing.

Therefore, four IMUs were deployed on the sagittal planes of the left and right thighs and shanks to construct a lower-limb perception system for the hip and knee joints, enabling the acquisition of kinematic information during climbing motions, as illustrated in Figure 1. The selected sensors are IM948 nine-axis attitude sensors (Manufactured by Chenyi Electronics Technology Co., Ltd. in Shenzhen, China). By capturing lower-limb motion data at each climbing stage, the system identifies the operator’s motion intention, where each movement phase is assigned a predefined intention label corresponding to specific climbing-related motion states.

Prior to feature extraction, raw IMU signals were preprocessed to improve data quality and consistency. First, a low-pass Butterworth filter was applied to remove high-frequency noise. Then, all signals were normalized using z-score normalization to ensure comparable scales across different channels and subjects. Subsequently, a sliding window segmentation strategy was adopted, where fixed-length windows with overlapping strides were used to divide continuous signals into short temporal segments. Feature extraction was performed within each window to generate the final input samples.

Therefore, this study focuses on an offline recognition framework for lower-limb movement intentions in the context of climbing ultra-high-voltage transmission and distribution towers. By employing parallel encoding from multiple IMUs and fusing hip and knee joint motion signals, the framework captures asymmetric and alternating periodic movements within the sequence. Subsequently, a temporal attention mechanism is introduced to focus on the recognition of short-term and weak features during critical climbing phases, and validation studies are conducted using real-world climbing data from 10 participants.

### 2.2. Model Architecture

**Figure 2:**
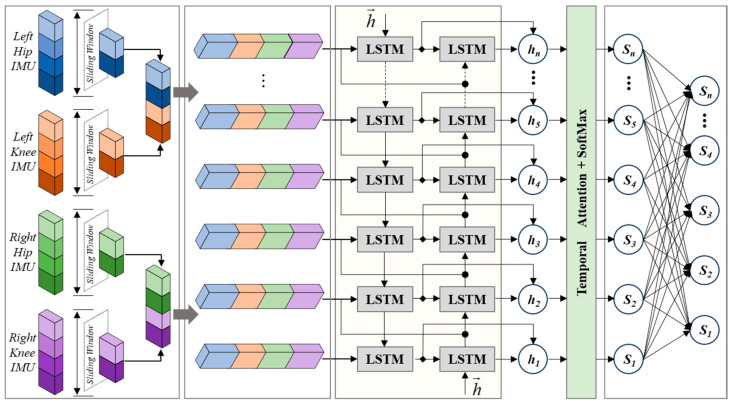
CNN–BiLSTM with Temporal Attention architecture for motion intention recognition of lower-limb exoskeletons in transmission tower climbing.

The architecture of the proposed motion intention recognition model is illustrated in Figure 2. The overall framework adopts a hierarchical design that integrates multi-source parallel processing and bidirectional temporal attention fusion, enabling end-to-end modeling from raw IMU signal inputs to motion intention classification outputs.

First, in the multi-source IMU data preprocessing module, four nine-axis IMU sensors are attached to the lower-limb segments, including the left thigh, left shank, right thigh, and right shank. Each IMU measures segmental motion, including tri-axial acceleration, angular velocity, and orientation. The thigh-mounted sensors primarily capture hip joint dynamics, while the shank-mounted sensors reflect knee joint movements, thereby providing a comprehensive representation of lower-limb kinematics during climbing.

To improve computational efficiency and reduce redundant information, this paper employs a task-driven feature selection strategy rather than directly using all nine sensor channels. Specifically, for each IMU, the three-axis acceleration and angular velocity are retained to characterize dynamic motion, while only the sagittal plane attitude angle is selected from the three attitude angles [25]. This is because the flexion and extension movements of the hip and knee joints during climbing primarily occur in the sagittal plane and are most relevant to the recognition of movement intent. Consequently, each IMU contributes 7 dimensions (3 accelerations, 3 angular velocities, and 1 sagittal plane angle), and the four IMUs together provide 28 dimensions of raw data. To mitigate the impact of varying action durations, a sliding window strategy is employed to segment the continuous signal into fixed-length, overlapping samples, thereby generating a standardized temporal input sequence.

Next, in the parallel BiLSTM temporal feature extraction module, the input sequences are organized into sensor-wise feature groups and fed into parallel bidirectional long short-term memory (BiLSTM) sub-networks. Each sub-network processes the temporal features from a specific IMU independently, preserving localized motion characteristics. The forward LSTM captures temporal dependencies in chronological order, while the backward LSTM processes the sequence in reverse to incorporate future context. The hidden states from both directions are concatenated to form bidirectional temporal representations.

Subsequently, in the temporal attention weighting module, the outputs from all BiLSTM branches are aggregated and refined through an attention mechanism. The importance of each time step is computed using a nonlinear scoring function, followed by Softmax normalization to generate adaptive attention weights. This mechanism enables the model to emphasize critical motion phases, such as limb lifting and load-bearing transitions, while suppressing less informative segments.

Finally, in the multi-intention classification module, the attention-weighted temporal features are fed into a fully connected layer followed by a Softmax classifier. The fully connected layer employs a ReLU activation function to enhance nonlinear representation capability, while Dropout regularization is applied to reduce the risk of overfitting. The model outputs a probability distribution over motion intention classes, and the class with the highest probability is selected as the final recognition result.

#### 2.2.1. Feature Extraction

To extract discriminative spatiotemporal features from raw IMU signals, a one-dimensional convolutional neural network (1D-CNN) is employed as the front-end feature extractor. Compared with handcrafted feature engineering methods, CNNs can automatically learn hierarchical representations from multi-channel inertial data and effectively capture local motion patterns. Given a sliding-window IMU segment,

(1) $$X\in {\mathbb{R}}^{TC}$$

where *T* denotes the window length and *C* represents the number of signal channels. The CNN encoder transforms *X* into high-level feature maps, enabling the extraction of abstract spatiotemporal representations from raw IMU sequences.

(2) $$Z^{\left(l\right)}=W^{\left(l\right)}*Z^{\left(l-1\right)}+b^{\left(l\right)}$$

(3) $$Z^{\left(0\right)}=X$$

(4) $$\mathrm{ReLU}\left(x\right)=\max\left(0,x\right)$$

where ∗ denotes the one-dimensional convolution operation, *W* represents the convolutional weights, and *b* is the bias term. ReLU(·) denotes the rectified linear unit activation function, which improves gradient propagation and enhances nonlinear representation capability. To accelerate convergence and ensure stable training performance, batch normalization is further introduced:

(5) $$\hat{Z}=\frac{Z-\mu }{\sqrt{\sigma^{2}+\varepsilon }}$$

where *μ* and *σ*^2^ denote the batch mean and variance, respectively, and *ε* is a small constant added for numerical stability. To reduce temporal resolution and enhance feature robustness, a max-pooling operation is further applied:

(6) $$P=\mathrm{MaxPool}\left(Z\right)$$

The features obtained after multiple convolutional layers are fed into the subsequent temporal modeling module as input, which can be expressed as

(7) $$F=\{f_{1},f_{2},\ldots ,f_{T}\},\;f_{t}\in {\mathbb{R}}^{d}$$

where *T* denotes the sequence length and *d* represents the feature dimension.

#### 2.2.2. Bidirectional LSTM Encoder for Temporal Modeling

**Figure 3:**
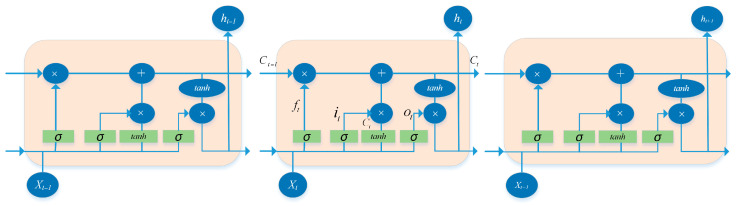
Structure diagram of LSTM.

To model long-range temporal dependencies in IMU motion sequences, the extracted feature representations are fed into a bidirectional long short-term memory (Bi-LSTM) encoder. Compared with unidirectional recurrent networks, the Bi-LSTM processes feature sequences in both forward and backward directions, enabling the model to exploit complete temporal contextual information within each motion segment. The architecture diagram of the forward LSTM model is shown in Figure 3. The forward LSTM processes the sequence from *t* = 1 to *T*, while the backward LSTM processes the sequence in reverse order:

(8) $$\begin{array}{l} \vec{h_{t}}=LSTM_{f}(f_{t},\vec{h_{t-1}})\\ \overset{\leftarrow }{h_{t}}=LSTM_{b}(f_{t},\overset{\leftarrow }{h_{t+1}}) \end{array}$$

At each time step, the internal operations of the LSTM unit are defined through the input gate, forget gate, output gate, candidate memory, cell state update, and hidden state output:

(9) $$\begin{array}{l} i_{t}=\sigma (W_{i}f_{t}+U_{i}h_{t-1}+b_{i})\\ f_{t}=\sigma (W_{f}f_{t}+U_{f}h_{t-1}+b_{f})\\ o_{t}=\sigma (W_{o}f_{t}+U_{o}h_{t-1}+b_{o})\\ {\tilde{c}}_{t}=\tanh(W_{c}f_{t}+U_{c}h_{t-1}+b_{c})\\ c_{t}=f_{t}⊙c_{t-1}+i_{t}⊙{\tilde{c}}_{t}\\ h_{t}=o_{t}⊙\tanh(c_{t}) \end{array}$$

where *σ*(·) denotes the Sigmoid activation function, tanh(·) represents the hyperbolic tangent function, and ⊙ indicates element-wise multiplication. $W_{*}$, $U_{*}$ and $b_{*}$ denote the learnable weight matrices and bias vectors, respectively. At each time step, the hidden states propagated in the forward and backward directions are concatenated to form a bidirectional representation. The final output sequence of the Bi-LSTM can be expressed as

(10) $$\begin{array}{l} h_{t}=\left[\begin{array}{c} \vec{h_{t}};\overset{\leftarrow }{h_{t}} \end{array}\right]\\ H=\{h_{1},h_{2},\ldots ,h_{T}\},\;h_{t}\in {\mathbb{R}}^{2d_{h}} \end{array}$$

where [;] denotes vector concatenation, and *d_h_* represents the number of hidden units in each directional LSTM. By introducing bidirectional temporal encoding, the model captures both historical and future contextual dependencies within each motion segment. This is particularly beneficial for lower-limb motion intention recognition in climbing scenarios, where transitional states depend not only on preceding actions but also on subsequent movements. Consequently, the Bi-LSTM encoder provides richer temporal representations for the subsequent attention weighting and classification modules.

#### 2.2.3. Temporal Attention Mechanism

Although the Bi-LSTM encoder can capture bidirectional temporal dependencies, not all time steps contribute equally to motion intention recognition. In climbing movements, discriminative information is often concentrated in short-duration events, such as foot-off, ladder contact, and support transitions. To emphasize these information-rich periods, a temporal attention mechanism is introduced to adaptively reweight the Bi-LSTM output sequence. Each hidden state is projected into an attention space:

(11) $$u_{t}=\tanh(W_{a}h_{t}+b_{a})$$

where *W*_*a*_ denotes the attention weight matrix, *b*_*a*_ represents the bias term, and $u_{t}$ is the intermediate attention representation. The normalized attention weights are then obtained through a Softmax function:

(12) $$\alpha_{t}=\frac{\exp(u_{t}^{⊤}u)}{\sum_{k=1}^{T}\exp(u_{k}^{⊤}u)}$$

where *u* denotes the attention context vector, and *α*_*t*_ represents the importance weight at time step *t*. The final temporal context representation is computed as the weighted sum of the hidden states, which captures the most informative temporal features:

(13) $$s=\sum_{t=1}^{T}\alpha_{t}h_{t},\;s\in {\mathbb{R}}^{2d_{h}}$$

The aggregated context vector is subsequently fed into the classifier:

(14) $$y=Softmax(W_{c}s+b_{c})$$

where $W_{c}$ and $b_{c}$ denote the classification parameters, and y represents the predicted probability distribution of motion intentions.

By assigning adaptive weights to different time steps, the temporal attention mechanism enables the model to focus on movement phases that are most relevant to intention discrimination. This design is particularly effective for recognizing short-duration and transitional climbing states, where critical kinematic cues may appear only within limited temporal intervals.

## 3. Experiments

### 3.1. Data Analysis

The experimental data was collected from records of 10 maintenance personnel qualified to perform high-altitude power line maintenance climbing operations whilst carrying out tasks on transmission towers. The dataset comprises 200 sequences of climbing movements, with a total duration of approximately 198 min. To prevent potential data leakage caused by overlapping sliding windows, the dataset was first divided at the subject level into training, validation, and test sets. Specifically, subjects were randomly assigned into three groups with a ratio of 7:2:1, ensuring that no data from the same subject appeared in multiple subsets. After the sequence-level split, the sliding time window strategy was implemented independently within each subset to segment continuous temporal signals into fixed-length standardized samples. This operation ensures that windowed segments from the same original motion sequence never appear in both training and test sets, thus avoiding overoptimistic performance caused by data leakage.

### 3.2. Experimental Environment

The experiments were conducted on a workstation equipped with an Intel Core i7-12700H CPU (14 cores, 20 threads, 2.7 GHz; Intel, Santa Clara, CA, USA), an NVIDIA RTX 3060 GPU with 6 GB of memory (NVIDIA, Santa Clara, CA, USA), 32 GB of DDR5 RAM, and a 1 TB SSD for storage. The software environment consisted of Ubuntu 22.04 as the operating system, Python 3.8 as the programming language, and PyTorch 1.12 as the deep learning framework. Data processing was performed using NumPy 1.23.5, Pandas 1.5.3, and SciPy 1.9.3, while Matplotlib 3.6.2 and Seaborn 0.12.2 were used for data visualization.

### 3.3. Evaluation Metrics

To train the proposed CNN–BiLSTM–Attention network for motion intention recognition, categorical cross-entropy loss was adopted as the primary optimization objective. Given the predicted probability distribution $\hat{y}$ and the ground-truth label vector *y*, the loss is defined as follows:

(15) $$L_{CE}=-\sum_{i=1}^{C}\hat{y_{i}}\log(y_{i})$$

where *y*_*i*_ denotes the predicted probability for class *i*, $\hat{y_{i}}$ is the ground-truth label, and *K* represents the total number of classes. Due to the significant variation in the duration of different actions during tower climbing, a weighted cross-entropy loss is introduced to mitigate class imbalance:

(16) $$\begin{array}{l} L_{WCE}=-\sum_{i=1}^{C}w_{i}{\hat{y}}_{i}\log(y_{i})\\ w_{i}=\frac{N}{C\cdot N_{i}}\\ L=\frac{1}{N}\sum_{n=1}^{N}L_{WCE}^{\left(n\right)} \end{array}$$

where *w*_*i*_ is the weight for class *i*, *N* denotes the total number of samples, and *N*_*i*_ represents the number of samples in class *i*. L is the overall optimization objective, and the model is trained by minimizing this loss function.

To comprehensively evaluate the model’s performance under multi-class and imbalanced data conditions, multiple evaluation metrics were employed, including accuracy, balanced accuracy, and F1 score.

(17) $$\begin{array}{l} Accuracy=\frac{\sum_{i=1}^{C}TP_{i}}{N}\\ BalAcc=\frac{1}{C}\sum_{i=1}^{C}\frac{TP_{i}}{TP_{i}+FN_{i}}\\ Precision_{i}=\frac{TP_{i}}{TP_{i}+FP_{i}}\\ Recall_{i}=\frac{TP_{i}}{TP_{i}+FN_{i}}\\ F1_{i}=\frac{2\times Precision_{i}\times Recall_{i}}{Precision_{i}+Recall_{i}} \end{array}$$

where ${TP}_{i}$ denotes the number of correctly predicted samples for class *i*, and *N* is the total number of samples. These metrics collectively assess overall classification accuracy, class-balanced performance, minority class recognition capability, and prediction consistency, providing a comprehensive evaluation of motion intention recognition in complex climbing scenarios.

## 4. Results

The hyperparameters of the proposed CNN–BiLSTM–attention network were determined empirically to achieve a balance between recognition accuracy and computational efficiency. Table 2 summarizes the detailed architectural and training configurations.

The CNN module adopts compact convolutional kernels to extract short-term kinematic variations, while the BiLSTM encoder leverages stacked bidirectional layers to strengthen long-range temporal dependency modeling. The attention dimensionality is configured to match the latent representation space, thereby facilitating effective temporal weight allocation. In addition, dropout regularization and a weighted loss formulation are incorporated to alleviate overfitting and class imbalance, respectively.

To ensure reliable and repeatable evaluation, all experiments were conducted with 10 independent runs, and the results are reported as mean ± standard deviation.

### 4.1. Overall Performance Comparison

To comprehensively evaluate the effectiveness of the proposed action intention recognition framework, we conducted 10 comparative experiments using the same experimental setup as our method against various baseline models. As shown in Table 3, the experimental results systematically compare the performance of traditional machine learning methods with representative deep learning methods.

**Figure 4:**
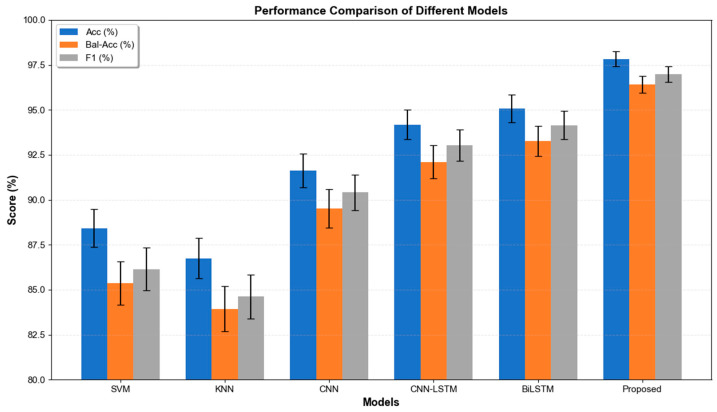
Comparison curves with other baseline models.

The proposed model achieved the best performance across all evaluation metrics. The visualization of the experimental results for the classification evaluation metrics is shown in Figure 4. Compared with the widely adopted CNN–LSTM architecture, the proposed framework improved classification accuracy by 3.65%, demonstrating the superiority of bidirectional temporal modeling and attention-based feature optimization.

### 4.2. Subject-Independent Performance (LOSOCV)

To further evaluate the generalization capability of the proposed model across different individuals, a subject-independent validation strategy was conducted using a leave-one-subject-out (LOSO) scheme. In each iteration, data from one subject were used as the test set, while data from the remaining subjects were used for training. All settings, including features, windowing parameters, and hyperparameters, are consistent with the experimental setup described earlier. The experimental results are summarized in Table 4.

The experimental results indicate that the proposed method achieves stable performance under cross-subject conditions, with only a slight reduction compared to the random split setting. This demonstrates the robustness and generalization capability of the model in practical applications. The performance gap between subject-dependent and subject-independent settings is mainly attributed to inter-subject variability in movement patterns and sensor placement, which will be further addressed in future work through domain adaptation and personalized modeling.

### 4.3. Ablation Study

To further verify the effectiveness and necessity of each component in the proposed model, a series of controlled ablation experiments were conducted. Specifically, several comparative architectures were designed, including single-channel LSTM, multi-channel LSTM (parallel), multi-channel BiLSTM, BiLSTM, BiLSTM with an attention mechanism, and parallel BiLSTM with attention and multi-feature fusion. The experimental results are summarized in Table 5.

**Figure 5:**
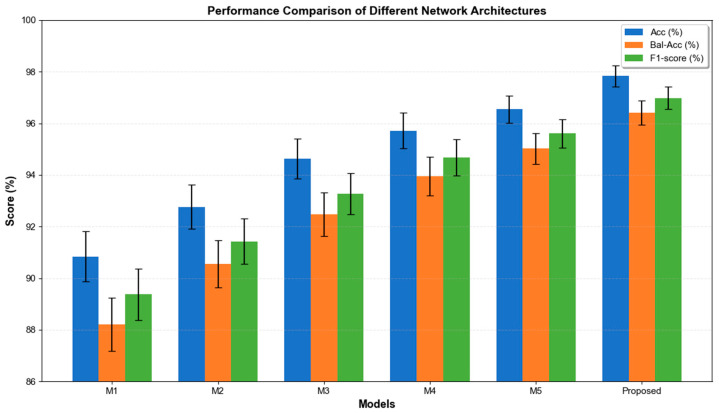
Ablation experiment results curves.

The visualization of the ablation experiment results is shown in Figure 5. By comparing M1 and M2, the introduction of multi-channel parallel processing increased the ACC from 90.84% to 92.76%, while the Bal-Acc improved by 2.34%, indicating enhanced robustness in recognizing minority motion states.

Compared with M2, M3 replaces the unidirectional LSTM with a BiLSTM encoder, yielding an ACC improvement of 1.87% and an F1 increase of 1.93%. This demonstrates that the forward LSTM captures historical dependency information, whereas the backward LSTM extracts future contextual cues; their bidirectional fusion strengthens the perception of non-periodic motion patterns.

A comparison between M3 and M4 shows that integrating both time-domain and frequency-domain statistical features further improves performance, with ACC and Bal-Acc increasing by 1.08% and 1.48%, respectively.

In addition, the results of M5 validate the necessity of incorporating the attention mechanism. The complete model achieves the best performance across all evaluation metrics, further confirming the necessity and effectiveness of each module design.

### 4.4. Confusion Matrix

**Figure 6:**
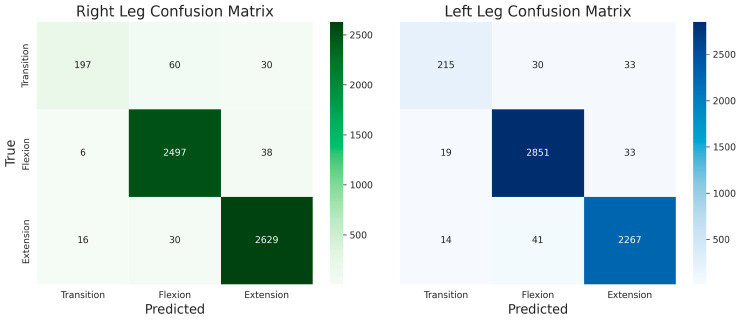
Confusion matrix results of lower-limb motion intention recognition during transmission tower climbing. Diagonal elements represent correct classifications, while off-diagonal elements denote misclassification counts.

To further analyze the recognition capability of the proposed model for lower-limb motion intention during transmission tower climbing, confusion matrices of the left and right lower limbs were constructed, as illustrated in Figure 6. In this figure, the vertical axis represents the true motion phases, while the horizontal axis denotes the model’s predicted results, enabling an intuitive visualization of classification accuracy across different action stages as well as their mutual misclassification relationships. By examining the proportion of diagonal elements and the distribution patterns of misclassified samples, the discriminative reliability of the model in key assistance phases—such as leg lifting, support, and stepping—can be further evaluated, along with the bilateral recognition consistency.

From the overall distribution, the confusion matrices of both the left and right lower limbs exhibit a pronounced diagonal dominance pattern, indicating that the proposed model achieves high classification accuracy across all motion phases. Among them, the flexion and extension phases demonstrate the most prominent recognition performance, with the highest proportion of samples concentrated along the diagonal. This suggests that the model can stably capture the inertial feature differences associated with joint motion during flexion/extension and load-bearing support processes.

In contrast, a small number of misclassifications are observed in the transition phase, as well as in flexion and extension phases when they occur within transitional intervals. These misclassifications primarily arise during the transition between flexion and extension. This phenomenon can be attributed to the short duration of the transition phase and the continuous, highly similar kinematic signal variations across adjacent motion states.

Further comparison between the left and right lower limbs reveals that the recognition distribution trends across all motion categories are largely consistent between the two sides, demonstrating the robustness and symmetry of the proposed model.

Therefore, accurately and rapidly mapping the classification results of the flexion, extension, and transition phases to the control of a lower-limb assistive exoskeleton for tower climbing is of great practical significance. When the exoskeleton detects that the hip and knee joints of the lower limbs are transitioning from other states to a flexed state, the exoskeleton actuators output positive torque; when the exoskeleton detects that the hip and knee joints of the lower limbs are transitioning from other states to an extended state, the exoskeleton actuators output negative torque; and when the exoskeleton detects that the hip and knee joints of the lower limbs are neither in a flexed nor an extended state, the actuators do not output torque, thereby reducing the physical exertion of maintenance personnel during tower climbing operations.

### 4.5. Classification Performance Evaluation

To further analyze and validate the proposed model’s capability in recognizing lower-limb motion intentions during transmission tower climbing—specifically flexion, extension, and the transitional state—Accuracy, Recall, and F1-score were calculated. The results are presented in Table 6.

The extension achieved the highest scores due to its longer duration and more stable inertial characteristics, whereas the flexion–extension exhibited slightly lower recall because of its shorter temporal span.

## 5. Conclusions

This study addresses the lower-limb exoskeleton assistance requirements in ultra-high-voltage transmission tower climbing operations by proposing a motion intention recognition model that integrates multi-source inertial sensing, bidirectional temporal modeling, and attention weighting. A convolutional neural network (CNN) is employed to extract local spatiotemporal features from multidimensional signals within sliding time windows, while a bidirectional long short-term memory network (BiLSTM) is introduced to effectively capture long-range temporal dependencies in non-periodic climbing motions.

A temporal attention mechanism is further incorporated to adaptively weight the contribution of features at different time steps, enhancing the representation of key motion phases—such as leg lifting and pedal thrust—while suppressing interference from transitional states and redundant information. Experimental results demonstrate that the proposed model outperforms conventional machine learning approaches and other deep learning architectures across multiple evaluation metrics, including accuracy, F1-score, and balanced accuracy.

Ablation studies validate the effectiveness of the multi-source parallel structure, bidirectional temporal encoding, and attention mechanism, providing reliable support for lower-limb exoskeleton assistive control. Future work will further integrate multimodal physiological and interaction signals—such as surface electromyography (sEMG) and plantar pressure—to explore lightweight embedded deployment and cross-subject adaptive learning methods, thereby enhancing engineering applicability and generalization performance in real-world power operation environments.

## Figures and Tables

**Table 1:** Description of lower-body movement during tower climbing.

Movement State	Definition
Extension	Perform leg-kicking movements with the hip and knee joints
Flexion	Perform leg-lifting and bending exercises for the hip and knee joints
Transition State	Other subtle dynamic transition phases between extension and flexion

**Table 2:** Hyperparameters and configuration details of the CNN–BiLSTM–attention network.

Module	Hyperparameter	Value/Setting
Input	IMU modality	Acc + Gyro + Sagittal angle
	Feature dimension	28
	Window length	10 (time steps)
	Window stride	5
	Butterworth low-pass filter	Cut-off frequency 20 Hz
	Time synchronization	100 Hz
	z-score Normalization mean	0
CNN Feature Extractor	Conv layers	2
	Kernel size	3
	Stride/Padding	1
	Filters	64 → 128
	Activation	ReLU
	Normalization	Batch Normalization
	Pooling	Max pooling
BiLSTM Encoder	Hidden units	128
	Layers	2
	Direction	Bidirectional
	Output dimension	256
Temporal Attention	Attention type	Additive attention
	Attention dimension	128
Fully Connected Layer	Neurons	128
	Activation	ReLU
	Dropout	0.2
Classifier	Output layer	Softmax
	Number of classes	3
Training Settings	Loss function	Weighted Cross-Entropy
	Optimizer	Adam
	Initial learning rate	0.001
	Batch size	64
	Epochs	100
	Early stopping	Patience = 15
	Learning rate decay	Step decay (0.5)
Efficiency Evaluation	Model parameters	1.8 M
	FLOPs	1.2 GFLOPs
	Single-window inference time	1.5 ms

**Table 3:** Comparative experimental results with other baseline models (mean ± std, * *p* < 0.05 vs. CNN-LSTM).

Model	Acc (%)	Bal-Acc (%)	F1 (%)
SVM	88.42 ± 1.06	85.37 ± 1.21	86.15 ± 1.18
KNN	86.75 ± 1.13	83.94 ± 1.27	84.62 ± 1.22
CNN	91.63 ± 0.94	89.52 ± 1.06	90.41 ± 0.98
CNN-LSTM	94.18 ± 0.82	92.11 ± 0.91	93.02 ± 0.87
BiLSTM	95.07 ± 0.76	93.26 ± 0.83	94.15 ± 0.79
Proposed	97.83 ± 0.41 *	96.41 ± 0.47 *	96.98 ± 0.43 *

**Table 4:** Subject-independent performance of the proposed method under LOSOCV (mean ± std).

Metric	Value (%)
Acc	95.26 ± 1.12
Ba-Acc	94.18 ± 1.23
F1	94.72 ± 1.18

**Table 5:** The ablation experimental results are presented (mean ± std).

Model ID	Architecture Description	Acc (%)	Bal-Acc (%)	F1-Score (%)
M1	Single-channel LSTM	90.84 ± 0.97	88.21 ± 1.03	89.37 ± 0.99
M2	Multi-channel LSTM (parallel)	92.76 ± 0.85	90.55 ± 0.92	91.42 ± 0.88
M3	Multi-channel BiLSTM	94.63 ± 0.78	92.48 ± 0.84	93.27 ± 0.80
M4	BiLSTM + Statistical Features	95.71 ± 0.69	93.96 ± 0.75	94.68 ± 0.71
M5	BiLSTM + Attention	96.54 ± 0.53	95.02 ± 0.59	95.61 ± 0.55
Proposed	All	97.83 ± 0.41	96.41 ± 0.47	96.98 ± 0.43

**Table 6:** Classification metric evaluation results (mean ± std).

Class	Precision (%)	Recall (%)
Transitional	94.12 ± 0.85	92.85 ± 0.91
Flexion	98.03 ± 0.42	97.41 ± 0.46
Extension	97.56 ± 0.45	96.88 ± 0.49

## Data Availability

The data presented in this study are available on request from the corresponding author.

## Abbreviations

The following abbreviations are used in this manuscript:

IMU	Inertial Measurement Unit
CNN	Convolutional Neural Network
LSTM	Long Short-Term Memory
BiLSTM	Bidirectional Long Short-Term Memory
GRU	Gated Recurrent Unit
RNN	Recurrent Neural Network
TCN	Temporal Convolutional Network
FC	Fully Connected
ReLU	Rectified Linear Unit
Softmax	Softmax Activation Function
Dropout	Dropout Regularization
